# Mitochondrial dysfunction increases pro-inflammatory cytokine production and impairs repair and corticosteroid responsiveness in lung epithelium

**DOI:** 10.1038/s41598-019-51517-x

**Published:** 2019-10-21

**Authors:** R. F. Hoffmann, M. R. Jonker, S. M. Brandenburg, H. G. de Bruin, N. H. T. ten Hacken, A. J. M. van Oosterhout, I. H. Heijink

**Affiliations:** 1 University of Groningen, University Medical Center Groningen, Department of Pathology and Medical Biology, Groningen, The Netherlands; 2 University of Groningen, University Medical Center Groningen, GRIAC Research Institute, Groningen, The Netherlands; 3 University of Groningen, University Medical Center Groningen, Department of Pulmonology, Groningen, The Netherlands

**Keywords:** Experimental models of disease, Molecular medicine, Chronic inflammation

## Abstract

COPD is characterized by chronic lung inflammation and irreversible lung tissue damage. Inhaled noxious gases, including cigarette smoke, are the major risk factor for COPD. Inhaled smoke first encounters the epithelial lining of the lungs, causing oxidative stress and mitochondrial dysfunction. We investigated whether a mitochondrial defect may contribute to increased lung epithelial pro-inflammatory responses, impaired epithelial repair and reduced corticosteroid sensitivity as observed in COPD. We used wild-type alveolar epithelial cells A549 and mitochondrial DNA-depleted A549 cells (A549 Rho-0) and studied pro-inflammatory responses using (multiplex) ELISA as well as epithelial barrier function and repair (real-time impedance measurements), in the presence and absence of the inhaled corticosteroid budesonide. We observed that A549 Rho-0 cells secrete higher levels of pro-inflammatory cytokines than wild-type A549 cells and display impaired repair upon wounding. Budesonide strongly suppressed the production of neutrophil attractant CXCL8, and promoted epithelial integrity in A549 wild-type cells, while A549 Rho-0 cells displayed reduced corticosteroid sensitivity compared to wild-type cells. The reduced corticosteroid responsiveness may be mediated by glycolytic reprogramming, specifically glycolysis-associated PI3K signaling, as PI3K inhibitor LY294002 restored the sensitivity of CXCL8 secretion to corticosteroids in A549 Rho-0 cells. In conclusion, mitochondrial defects may lead to increased lung epithelial pro-inflammatory responses, reduced epithelial repair and reduced corticosteroid responsiveness in lung epithelium, thus potentially contributing to the pathogenesis of COPD.

## Introduction

Chronic obstructive pulmonary disease (COPD) is a complex disease with increasing morbidity and mortality worldwide, characterized by irreversible airflow limitation and accelerated lung function decline. COPD is mainly caused by noxious environmental factors, including cigarette smoke, resulting in exaggerated lung inflammatory responses. Abnormal tissue repair upon cigarette smoke-induced inflammation and damage is an important pathophysiologic feature of COPD, leading to fibrosis in the small airways and/or destruction of lung tissue (emphysema). Despite their broad anti-inflammatory effects, inhaled corticosteroids (ICS) provide relatively little therapeutic benefit in COPD. They reduce exacerbations, but do not effectively change the course of the disease nor the tissue damage that results from chronic airway inflammation^1–5^. Furthermore, we recently observed that airway epithelial cells from COPD patients display reduced responsiveness to corticosteroids^2^.

Inhaled cigarette smoke first encounters the epithelial lining of the lungs, where it induces oxidative stress. This inflicts mitochondrial dysfunction and cellular damage in lung epithelium, promoting the release of cytokines that attract and activate neutrophils, e.g. CXCL8^6^. In addition, oxidative stress has been implicated in corticosteroid unresponsiveness^1,7^. Mitochondria are major intracellular targets of oxidative stress. Our previous findings indicate that bronchial epithelial cells from COPD patients display mitochondrial abnormalities, with depletion of cristae, increased expression of mitochondrial stress marker PINK1 and persistent mitochondrial damage. Similar mitochondrial changes were observed in epithelial cells that were exposed to cigarette smoke *in vitro* for 6 months, which was accompanied by increased pro-inflammatory activity^8^. In addition, increased PINK1 expression has been observed in alveolar epithelium of COPD patients^9^. However, it is currently unknown whether mitochondrial dysfunction contributes to aberrant epithelial pro-inflammatory activity and damage and repair responses in COPD. Of interest, Islam *et al*. have shown that transfer of intact mitochondria by mesenchymal stem/stromal cells can contribute to lung tissue repair in a mouse model of lethal lung injury, suggesting that intact mitochondria may play be crucial for lung regeneration and that loss of mitochondrial function may impair this process^10^. Furthermore, loss of mitochondrial function is known to lead to metabolic reprogramming with a switch to glycolysis, which has been implicated in corticosteroid resistance in lymphoblastic leukemia^11,12^. The causal role of metabolic reprogramming in the pathogenesis of COPD is not well known. Therefore, our aim was to investigate whether mitochondrial dysfunction is sufficient to induce epithelial abnormalities as observed in COPD.

We hypothesized that mitochondrial dysfunction has important implications for lung epithelial responses, leading to increased pro-inflammatory activity, altered ICS responsiveness and impaired epithelial repair responses. To address our hypothesis, we compared wild-type human alveolar A549 cells and A549 cells with depleted of functional mitochondria, A549 Rho-0. We show that A549 Rho-0 produce higher levels of pro-inflammatory cytokines, are less responsive to corticosteroids and display impaired repair responses compared to wild-type A549 cells.

## Material and Methods

### Cell culture

The wild type alveolar carcinoma cell line A549 and mitochondria-depleted A549 Rho-0 cells were kindly provided by Dr. Lodovica Vergani (Padova University, Padova, Italy), created and characterized as described previously^13,14^ by culturing A549 in addition of 50 ng/mL Ethidium Bromide for 8–12 passages. Cells were cultured in Dulbecco’s Modified Eagle’s medium (DMEM, Sigma, St. Louis, MO, (D6429-500ML) supplemented with MEM Amino Acids (50×) solution (Sigma, M7020-100ML), MEM Non-essential Amino Acid Solution, (Sigma, M7145-100ML), vitamins (Sigma, M6895-100ML) sodium pyruvate, uridine 50 ng/ml (Sigma, cat. n. U-3003), 2,5 µg/ml amphotericin (Sigma-A2942-100ML), 25% foetal bovine serum (FBS; Hyclone, Logan, UT), 100 U/ml penicillin and 100 µg/ml streptomycin (Invitrogen (Gibco), Breda, The Netherlands) in uncoated T25 flasks. Before experimentation, cells were grown ∼90% confluence and serum-deprived overnight.

### PCR

RNA was isolated from cells or lung tissue using TRIzol. Samples were treated with RNAse Free DNAse and subsequently cleaned with RNeasy Mini Kit (Qiagen, Valencia, CA). cDNA was synthesized with the iScript cDNA Synthesis Kit (BioRad, Hercules, CA). Gene expression was analyzed by real-time PCR using the Taqman® according to the manufacturer’s guidelines (Applied Biosystems, Foster City, CA). the TaqMan Master Mix and validated probes for *NADH dehydrogenase (complex I)*, *cytochrome C oxidase (complex IV) subunit III* and *ATPase subunit F1α (complex V)*, and the housekeeping genes *β*_2_*-microglobulin (β*_2_*µG)* and *Peptidylprolyl isomerase A (PPIA)* were purchased from Applied Biosystems. Relative mRNA expression was normalized to the mean expression of the housekeeping genes (2^−ΔCt^).

### Western blotting

Cell lysates were prepared and immunodetection was performed as described previously^15^ using MitoProfile total OXPHOS antibody cocktail (Mitosciences, Eugene OR) anti-Mn-SOD (EMD Millipore Corporation, Billerica MA) and anti-GAPDH (Cell Signalling Technology, Danvers MA, USA) as loading control. Densitometry was performed using the gel-scan program QuantityOne.

### ATP assay

Intracellular ATP levels of A549 were measured after ATP extraction. To extract ATP, cells were lysed using 0.5% TCA. Subsequently, TCA was neutralized with TE-buffer. ATP was measured with the luciferin–luciferase method (Enliten ATP assay system, Promega). Briefly, 100 μl sample was added to 50 μl of ATP assay mix and the luminescence was measured with a Luminoskan® Ascent microplate luminometer (Thermo Scientific, Waltham, USA) for 1 hour.

### Lactate assay

Lactate levels were measured in cell-free culture supernatants (24 hours) using a lactate assay kit (BioVision, Milpitas, USA) according to manufacturer’s protocol.

### Cytokine levels

Cell-free culture supernatants (24 hours) were collected and analysed for CXCL8 using a duo-set ELISA assay (R&D Systems Europe, Abingdon, UK) or analysed for CCL20, CXCL10 (IP-10), CCL2 (MCP-1), CCL3 (MIP-1α), CCL4 (MIP-1β), CCL5 (RANTES), G-CSF, IL-6 and IL-12 using a multiplex ELISA (Millipore, Billerica, MD) following manufacturer’s instructions.

### ECIS

Electrical resistance properties of confluent or wounded cells were measured using Electric Cell-substrate Impedance Sensing (ECIS, Applied Biophysics, Troy, NY, USA) as described previously^16^. Upon inoculation in ECIS arrays, cells were incubated with/without 10 nM budesonide for 48 hours and resistance and capacitance were measured at 400 Hz and 40 kHz respectively. Cells were wounded by electroporation (5 V, 40 kHz, 30 s) upon establishment of a confluent monolayer.

### Confocal microscopy

Cells were stained with JC1, MitoTracker® DeepRed FM and dsDNA stain Picogreen (Molecular Probes, Invitrogen) for 45 minutes in D-PBS (GIBCO, 14287-080) according to manufacture protocol. Subsequently, cells were washed twice with D-PBS and visualized using a Leica AOBS confocal microscope.

### Statistical analysis

Differences between the wild type and Rho-0 cells were evaluated by the Mann-Whitney test, 2-way ANOVA for time curves or the Wilcoxon signed rank for differences within the cell lines, as indicated in the figure legends. P = < 0.05 was considered statistically significant.

## Results

### Depletion of mitochondrial (mt)DNA and characterization of mitochondrial dysfunction in A549 Rho-0 compared to wild-type A549 cells

**Figure 1:**
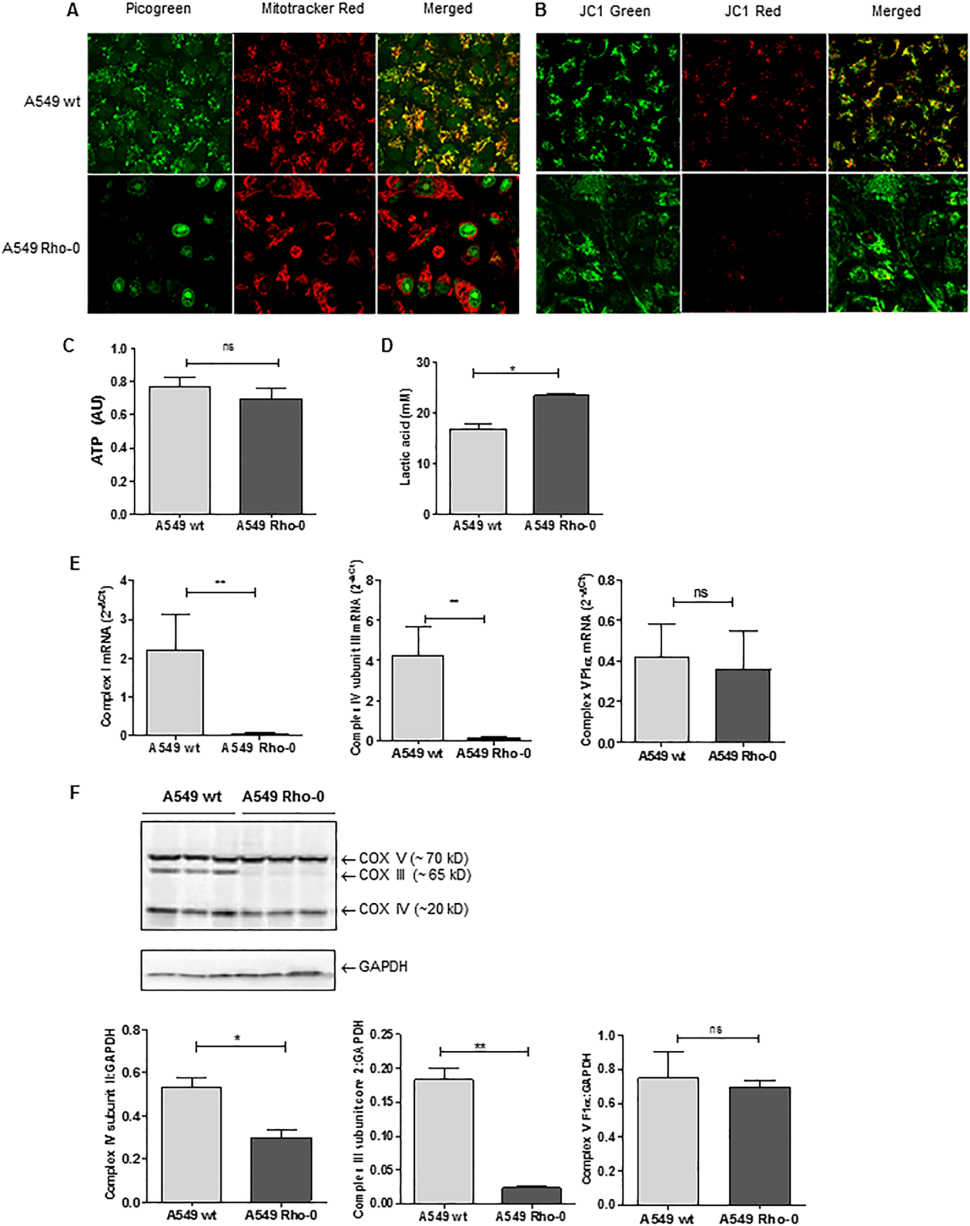
Mitochondrial (mt)DNA depletion and mitochondrial dysfunction in A549 Rho-0 cells. A549 wild-type (wt) and Rho-0 cells were grown to confluence and serum deprived for 24 hrs. (**A**) Cells were stained with Picogreen for detection of the DNA and Mitotracker DeepRed. Co-localization of DNA and mitochondrial bodies indicates mtDNA. Representatives of 3 independent experiments are shown. (**B)** Cells were stained with JC1 staining mitochondrial membrane potential is lowered by depletion of mtDNA in the A549 Rho-0 cells when compared to control cells. (**C)** ATP (mean ± SEM, n = 6–7) and (**D)** lactate (mean ± SEM, n = 3) were measured in cell-free culture supernatants. (**E)** RNA was isolated, cDNA synthesized, mRNA expression of *NADH dehydrogenase (complex I), cytochrome c oxidase (complex IV) subunit III* and *ATPase subunit F1α (complex V)* analyzed by qPCR and related to the expression of housekeeping genes *β2µG* and *PPIA*. **(F)** Cell lysates were prepared and complex III, Complex IV and the ATPase subunit components were detected by western blotting, analysed by densitometry and related to GAPDH (mean ± SEM, n = 3). *p < 0.05 and **p < 0.01 between the indicated values as measured by the one-tailed Mann-Whitney test.

First, we assessed whether mtDNA was successfully depleted in the A549 Rho-0 cells. We analyzed both wild-type A549 and A549 Rho-0 cell lines for the presence of mtDNA and mitochondrial bodies. Co-localization of DNA (Picogreen) and mitochondrial bodies (Mitotracker DeepRed) was clearly visible in wild-type A549, but not in A549 Rho-0 cells, confirming the lack of mtDNA (Fig. 1A). JC1 staining showed a drastic decrease in mitochondrial membrane potential the A549 Rho-0 cells (Fig. 1B). Surprisingly, the intra-cellular concentrations of ATP did not differ between the wild-type A549 and the A549 Rho-0 cells, indicating an alternative pathway for ATP generation than the mitochondrial-mediated oxidative phosphorylation (OXPHOS) process in A549 Rho-0 cells (Fig. 1C). Loss of mitochondrial function may lead to compensatory secondary metabolism, glycolysis, to produce ATP as well as lactate. To confirm a glycolytic switch in the A549 Rho-0 cells, we analyzed lactate production. Indeed, lactate levels in the culture medium of A549 Rho-0 cells were significantly increased when compared to wild-type A549 cells (Fig. 1D). This glycolytic reprogramming was further supported by a significant reduction in the mRNA and/or protein expression of specific components of the OXPHOS system, complex I, complex III subunit core 2 and complex IV subunit 2,3 (Fig. 1E,F). The expression of nuclear-encoded ATPase subunit α was not affected, indicating that only mitochondrial DNA was depleted in the Rho-0 cells. As activation of the redox sensitive PI3K/Akt pathway has been implicated in the switch to glycolysis, we studied phosphorylation of Akt and observed higher phospho-Akt levels in Rho-0 compared to wild-type A549 cells, in further support of the glycolytic switch (Supplementary Fig. 1).

### Elevated pro-inflammatory cytokine/chemokine secretion in A549 Rho-0 cells

**Figure 2:**
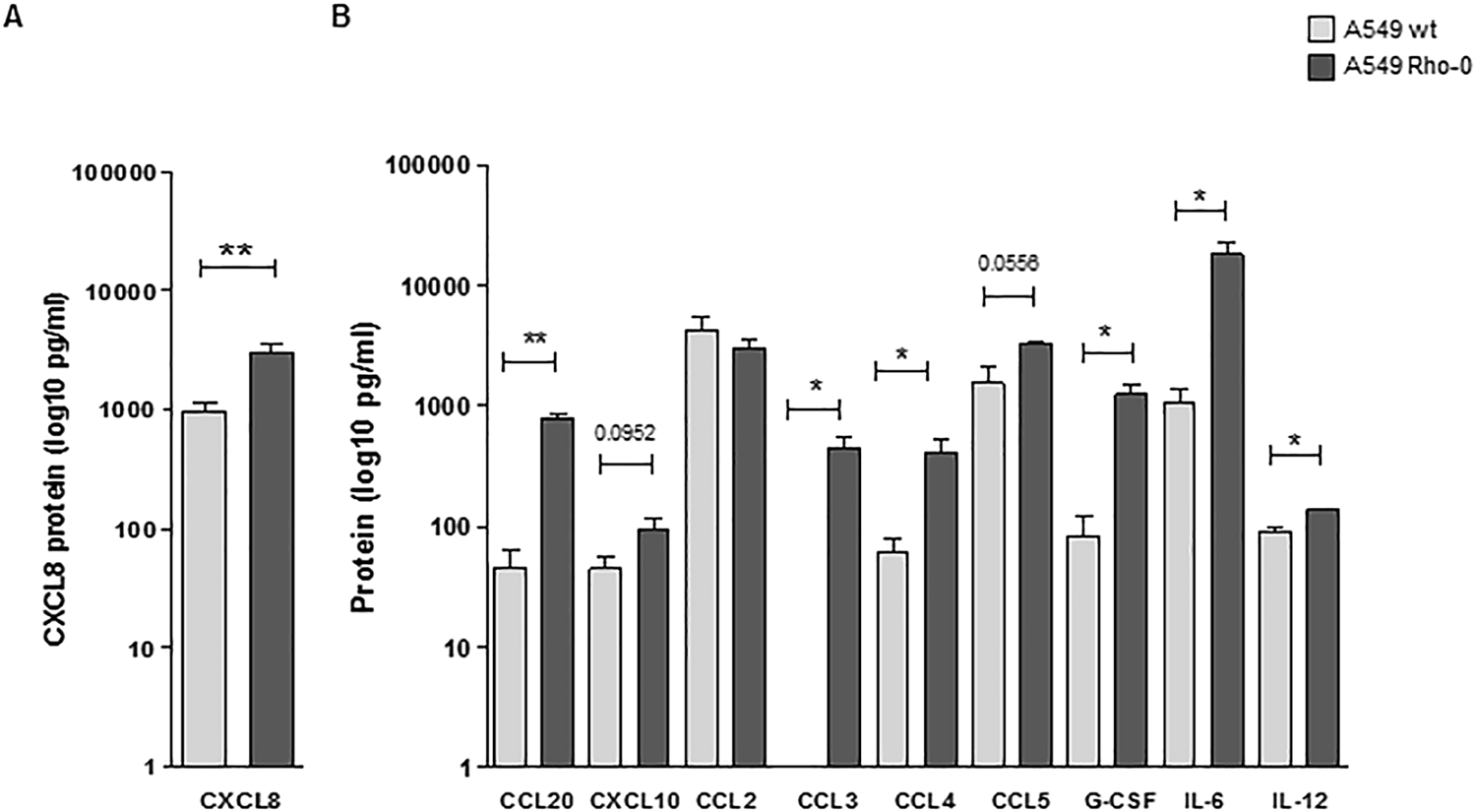
Increased pro-inflammatory activity in A549 Rho-0 compared to wild-type A549 cells. A549 wild-type (wt) and Rho-0 cells were grown to confluence, serum deprived overnight and supernatants were collected 24 hours later. (**A)** CXCL8 (mean ± SEM, n = 5) and (**B)** CCL20, CXCL10, CCL2, CCL3, CCL4, CCL5, G-CSF, IL-6 and IL-12 (mean ± SEM, n = 5) levels (pg/ml) were measured in cell-free supernatants using ELISA and multiplex ELISA respectively. *p < 0.05 and **p < 0.01 between the indicated values as analysed by the two-tailed Mann-Whitney test.

Next, we studied the effect of mitochondrial dysfunction on the pro-inflammatory cytokine response of lung epithelial cells. We measured a panel of pro-inflammatory epithelial cytokines/chemokines that have been associated with lung inflammation in COPD patients and/or show increased levels in COPD lungs, i.e. CXCL8^17,18^, CCL20^19,20^, CXCL10 (IP10)^21–23^, CCL2^24^, CCL3^25^, CCL4, CCL5 ^26^, G-CSF^27^, IL-6^28,29^ and IL-12^21,30,31^. Of these, the secretion of CXCL8 (Fig. 2A), CCL20, CCL3, CCL4, G-CSF, IL-6 and IL-12 was significantly higher in Rho-0 compared to wild-type A549 cells (Fig. 2B), while baseline levels of CXCL8 in wild-type A549 cells were within the same range as in primary airway epithelial cells^2^. A trend towards higher levels was observed for CCL5 and CXCL10, while CCL2 secretion was not different between A549 wild-type and Rho-0 cells (Fig. 2B). Collectively, these data suggest that mtDNA depletion results in an increased pro-inflammatory response in lung epithelial cells.

### Reduced repair capacity in A549 Rho-0 cells

**Figure 3:**
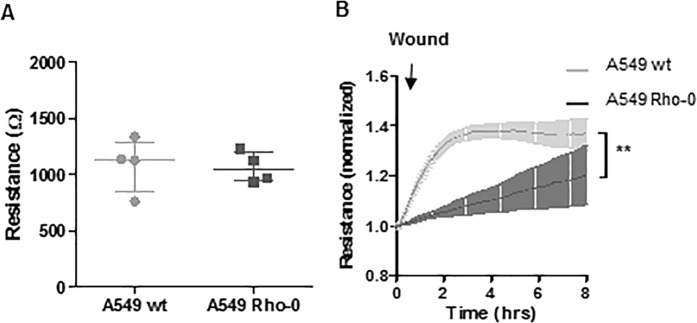
A549 Rho-0 cells display reduced wound repair compared to wild-type A549 cells. (**A)** A549 wild-type and Rho-0 cells were seeded in a density of 100.000 cells/well in duplicates in ECIS arrays and grown to confluence for 24 hours. Resistance was measured at a frequency of 400 Hz (mean ± SEM, n = 4). (**B)** Confluent monolayers were wounded by electroporation. Resistance was monitored for 8 hours at 400 Hz and levels were normalized to the values immediately after wounding (mean ± SEM, n = 4). **p < 0.01 between the indicated values as analyzed by 2-way ANOVA.

In addition to pro-inflammatory responses, we studied whether mitochondrial dysfunction impacts on the integrity of the epithelial monolayer and its recovery upon injury by electroporation using electric cell-surface impedance sensing (ECIS). We assessed A549 integrity by the build-up of resistance upon growth to confluence, which we previously demonstrated to depend more strongly on cell-substrate contacts than on formation of intracellular junctions in A549 cells using ECIS^16^. We did not observe a significant difference between A549 wild-type and Rho-0 cells in absolute resistance values upon establishment of a confluent monolayer (Fig. 3A). Next, we assessed the ability to recover the monolayer upon wounding by electroporation, requiring epithelial migration to repopulate the damaged area, as measured by the increase in resistance^16^. Wild-type A549 were able to recover from this type of wounding within 2–4 hours, as indicated by the stabilization of the resistance levels (Fig. 3B). The repair response of A549 Rho-0 cells was attenuated, taking approximately two times longer than wild-type A549 cells to recover, as reflected by delayed stabilization of resistance values (Fig. 3B). This suggests that mtDNA depletion reduces epithelial repair capacity specifically after injury.

### A549 Rho-0 cells display reduced corticosteroid responsiveness

**Figure 4:**
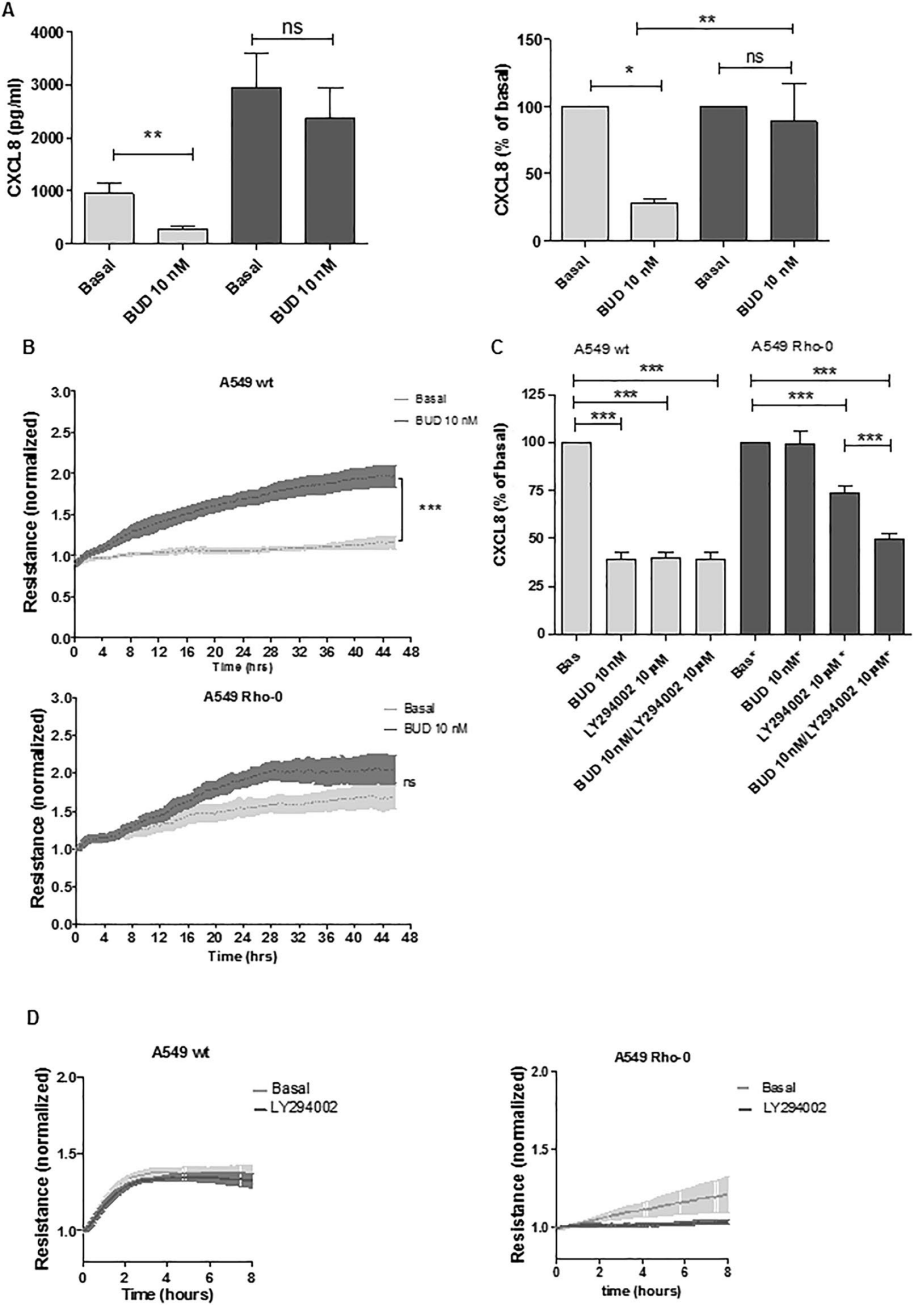
A549 Rho-0 cells are less sensitive to budesonide than wild-type A549 cells, which is restored by blocking of PI3K/Akt signaling. (**A)** A549 wild-type and Rho-0 cells were grown to confluence, serum deprived overnight and incubated with/without budesonide (BUD, 10 nM) for 24 hours. CXCL8 was measured in cell-free supernatant and expressed as absolute values and as percentage of the levels without BUD (mean ± SEM, n = 56). (**B)** A549 wild-type and Rho-0 cells were grown to confluence in ECIS arrays for 24 hours. Subsequently, 10 nM budesonide (BUD) or vehicle was added and cells were cultured for another 48 hours. Resistance was monitored at 400 Hz and normalized to the levels immediately after the addition of BUD (mean ± SEM, n = 4). (**C)** A549 wild-type and Rho-0 cells were grown to confluence, serum deprived overnight and incubated with/without budesonide (BUD, 10 nM) for 24 hours. LY294002 (10 µM) was added 30 min before the exposure to BUD. CXCL8 was measured in cell-free supernatant. CXCL8 levels (mean ± SEM, n = 5) are expressed as percentage of the levels without BUD. (**D)** A549 wild-type and Rho-0 cells were grown in ECIS arrays. Confluent monolayers were wounded by electroporation. LY294002 (10 µM) was added 60 min prior to wounding. Resistance was monitored for 8 hours at 400 Hz and levels were normalized to the values immediately after wounding (mean ± SEM, n = 4). *p < 0.05, **p < 0.01 ***p < 0.001 between the indicated values as assessed by the Wilcoxon signed rank test within cell lines, the Mann Witney test between cell lines, 1-way ANOVA with Bonferroni’s multiple comparison test for panel c, 2-way ANOVA for ECIS data.

To investigate whether mitochondrial dysfunction also reduces the responsiveness to corticosteroids, as has been observed in COPD and for CXCL8 production in airway epithelial cells upon exposure to oxidative stress^32^, we studied the suppressive effect of the ICS budesonide on CXCL8 secretion in wild-type A549 and A549 Rho-0 cells. We observed that budesonide significantly suppressed CXCL8 secretion by approximately 75–80% in wild-type A549 cells. Of interest, budesonide failed to significantly inhibit CXCL8 secretion in A549 Rho-0 cells (Fig. 4A). This suggests that the pro-inflammatory response triggered by mitochondrial dysfunction triggers is insensitive to corticosteroids.

In addition to the suppressive effects on cytokines, we have previously reported that corticosteroids exert protective effects on epithelial barrier function^2^. Therefore, we tested whether mitochondrial dysfunction also impairs the responsiveness to ICS with respect to effects on epithelial integrity. We monitored resistance as measure of epithelial integrity in the presence and absence of budesonide^16^. The presence of budesonide significantly increased the resistance in wild-type A549 cells, while A549 Rho-0 cells were again less responsive to budesonide, which was not able to significantly resistance in these cells (Fig. 4B).

Next, we studied whether the observed switch to glycolysis and activation of the associated redox sensitive PI3K/Akt signaling pathway is involved in the observed ICS insensitivity in A549 Rho-0 cells, as PI3K has previously been implicated in corticosteroid unresponsiveness in COPD^33^. We blocked PI3K/Akt activity using the specific inhibitor LY294002 (10 μM). In both wild-type A549 and A549 Rho-0 cells, we observed a marked reduction in CXCL8 secretion when the PI3K pathway was blocked (Fig. 4C). LY294002 was not able to further reduce CXCL8 secretion in A549 Rho-0 cells when budesonide was present, indicating that budesonide maximally inhibited CXCL8 expression. Of note, inhibition of the PI3K/Akt pathway restored the sensitivity to ICS in A549 Rho-0 cells. In contrast to the absence of LY294002, budesonide significantly reduced CXCL8 secretion in A549 Rho-0 cells in the presence of LY294002 (Fig. 4C). Thus, our data suggest that the mitochondrial defect in A549 Rho-0 cells induces PI3K/Akt-mediated corticosteroid insensitivity in lung epithelial cells. Finally, we studied the effect of PI3K/Akt inhibition on the repair capacity of A549 Rho-0 cells. In the presence of LY294002, A549 Rho-0 cells were unable to recover from wounding, whereas LY294002 did not affect the ability to recover from wounding in wild-type A549 cells (Fig. 4D). This indicates that Rho-0 cells are dependent on PI3K activity for their repair response, while this is not the case for wild-type cells, further confirming the reliance on PI3K-dependent glycolysis in A549 Rho-0 cells.

## Discussion

We show for the first time that human alveolar epithelial cells lacking functional mitochondria display increased production of pro-inflammatory cytokines and impaired repair responses. This is accompanied by reduced responsiveness to corticosteroids, which may be mediated by activation of the glycolysis. While ATP production was not impaired in A549 cells with dysfunctional mitochondria, we observed higher levels of lactate, and inhibition of the glycolysis-associated PI3K pathway restored corticosteroid sensitivity in A549 Rho-0 cells.

We previously observed that long-term cigarette smoke-exposed bronchial epithelial cells as well as bronchial epithelial cells from COPD patients display mitochondrial abnormalities^8^. These together with our current findings may help to explain the reduced corticosteroid responsiveness in bronchial epithelial cells from COPD patients compared to those from smoking and non-smoking controls^2^. Moreover, airway epithelial cells from COPD patients have been reported to secrete higher levels of CXCL8 than epithelial cells from controls^34^, which may also be related to mitochondrial dysfunction. Our previous data show that mitochondrial dysfunction is accompanied by increased secretion of the pro-inflammatory cytokines CXCL8 and IL-6^8^. Here, we show that the depletion of functional mitochondria leads to increased levels of CXCL8 and IL-6 as well as CCL20, G-CSF, CCL3 and CCL4, and IL-12, all pro-inflammatory cytokines that were found to be increased in lungs of COPD patients^17–21,24,25,27–31,35^.

The effect of depletion of functional mitochondria on these pro-inflammatory cytokines/chemokines was not a general effect on cytokine release, as for instance the secretion of CCL2 was not affected by depletion of mtDNA. Therefore, activation of specific pathways and/or transcription factors is more likely involved. Our findings may have important implications, as CXCL8 is a well know chemoattractant for neutrophils, which produce reactive oxygen species (ROS) and various proteases such as neutrophil elastase to damage the mitochondria and directly cause alveolar tissue damage in a positive feedback loop.

When depleted of mtDNA, cells cannot perform normal electron transport for ATP synthesis and rely on ATP derived from glycolysis, where glucose is metabolized for survival and growth to produce lactate^36^. This metabolic reprogramming is dependent on the activation of PI3K signaling, as demonstrated in naive T cells and cancer cells^37,38^. Of interest, it has previously been reported that corticosteroid resistance in T-lineage acute lymphoblastic leukemia is associated with the upregulation of glycolysis and activation of PI3K/Akt/mTOR signaling^11,12^. We observed a switch to glycolysis in Rho-0 cells, as indicated by increased lactate production and reduced expression of specific OXPHOS components. Furthermore, we found that inhibition of PI3K reversed the ICS unresponsiveness of CXCL8 production in A549 Rho-0 cells. PI3K inhibition itself significantly reduced CXCL8 production, and the insensitivity of CXCL8 to ICS in A549 Rho-0 cells could thus be a consequence of PI3K activity being insensitive to corticosteroids, resulting in corticosteroid-insensitive CXCL8 production. Alternatively, the increased PI3K activity may have resulted in general corticosteroid responsiveness, e.g. by mechanisms involving glycogen synthase kinase (GSK)-3β inactivation and post-translational histone deacetylase 2 (HDAC2) modifications and proteasomal degradation of HDAC2, leading to altered acetylation of the corticosteroid receptor and/or histones within the promoter regions of pro-inflammatory genes^3,39,40^. We previously demonstrated that IL-17 reduces ICS responsiveness of CXCL8 production in airway epithelial cells in a PI3K and HDAC2-dependent manner^41^. Similar mechanisms may be involved corticosteroid unresponsiveness of CXCL8 upon mtDNA depletion, although further studies will be required to establish this. Cells depleted of functional mitochondria were also less sensitive to ICS with respect to effects on the integrity of the epithelial monolayer, supporting the notion of general corticosteroid unresponsiveness in these cells. Since ICS fail to efficiently suppress inflammation in the majority of COPD patients, it will be of interest to elucidate the precise mechanisms of corticosteroid unresponsiveness in mitochondria-depleted epithelial cells in future studies.

**Figure 5:**
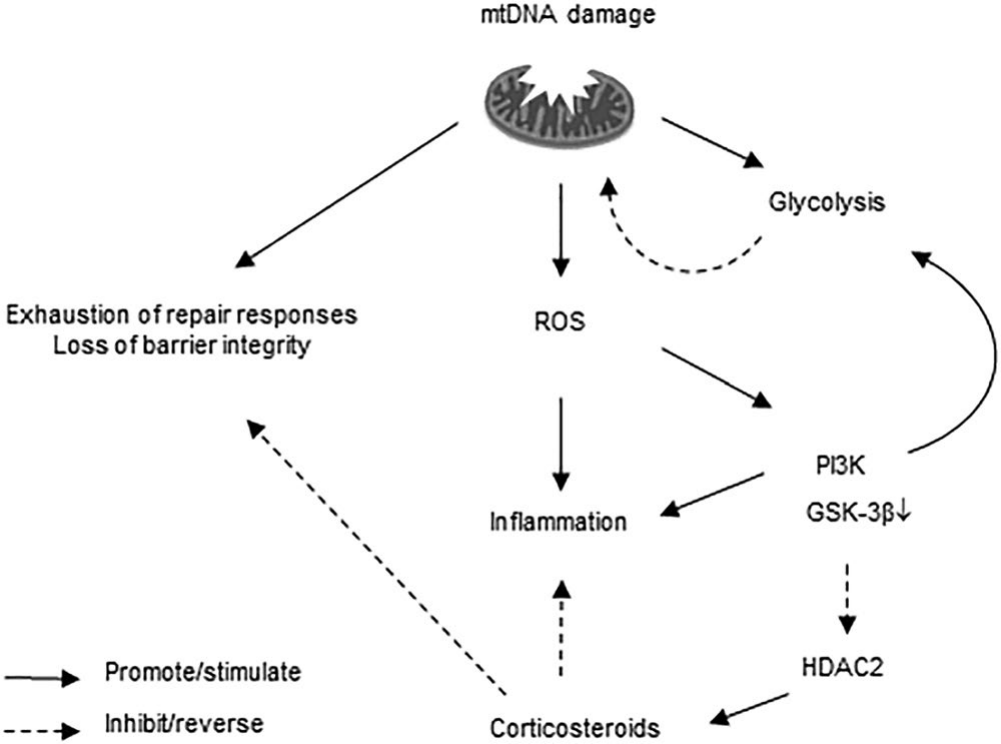
Schematic of proposed processes upon mitochondrial DNA (mtDNA) depletion in lung epithelial cells. During homeostasis, ATP is produced by oxidative phosphorylation and there is a balance between active and inactive PI3K/GSK-3βsignaling, ensuring limited glycolysis. Upon mitochondrial damage/dysfunction induced by mtDNA depletion, increased release of reactive oxygen species (ROS) production results in increased activity of redox sensitive kinases such as PI3K and a subsequent increase in production of pro-inflammatory mediators. Cells can no longer perform normal electron transport for ATP synthesis and rely on ATP derived from glycolysis, which is accompanied by impairment/exhaustion of repair responses. This glycolytic switch is promoted by the activation of PI3K signaling and subsequent inactivation of GSK-3β and HDAC2, resulting in reduced corticosteroid sensitivity of pro-inflammatory responses as well as reduced potential of corticosteroids to improve barrier integrity, the latter through mechanisms that need further elucidation.

In addition to the effects on pro-inflammatory responses and corticosteroid responsiveness, our findings suggest that mitochondrial dysfunction has implications for lung tissue repair in COPD. We observed that mitochondrial depletion impairs the ability of the A549 cells to repair upon injury. Although we did not further study the mechanisms by which functionally intact mitochondria promote epithelial regeneration, it is conceivable that epithelial migration to cover the wounded area requires active energy metabolism. Metabolic reprogramming may thus be involved in the impaired epithelial regeneration that is thought to contribute to the pathogenesis of the disease. In line with such a role for mitochondrial dysfunction in the pathogenesis of COPD, Mizumura and co-workers have shown that cigarette smoke causes mitochondrial dysfunction in a mouse model of COPD, while *Pink1* deficient mice were protected from mitochondrial dysfunction and airspace enlargement upon cigarette smoke exposure^9^. Of note, lung ageing is also associated with mitochondrial dysfunction, accompanied by exhausted repair capacity of stem/progenitor cells and emphysema-like features^8,42–44^. In contrast to the detrimental effect of PI3K/Akt signaling with respect to cytokine production and corticosteroid responsiveness, the inhibition of PI3K completely abolished the ability of A549 Rho-0 cells to recover from wounding. These data indicate that different mechanisms are responsible for abnormal inflammatory and impaired repair responses upon mitochondrial dysfunction and confirm that Rho-0 cells rely on PI3K-dependent activation of glycolysis for metabolic activity involved in repair responses (Fig. 5).

Our current findings are limited to the use of a cell line, since the depletion of mtDNA for creating Rho-0 cells requires long-term passaging of cells during exposure to a low dose of ethidium bromide. Moreover, A549 cells are cancerous and it is worth mentioning that metabolic pathways may be shifted to some extent in carcinoma cells at baseline, an effect known as the Warburg effect. Therefore, in future studies, it will be of interest to test the effect of mitochondrial inhibitors in non-cancerous primary epithelial cells.

In conclusion, our data indicate that mitochondrial dysfunction leads to increased pro-inflammatory activity, inefficient repair and reduced responsiveness to GCs in alveolar epithelium. Our results suggest that novel strategies towards improved mitochondrial function may be promising in COPD. Furthermore, restoration of steroid responsiveness, e.g. by means of pharmacological inhibition of the PI3K pathway, may increase the beneficial effects of corticosteroids on lung epithelial cells in COPD.

## Supplementary information

Supplementary methods and figure 1

## Data Availability

The datasets generated during the current study are available from the corresponding author on reasonable request.
