# Supplementary material for: Mitochondrial dysfunction increases pro-inflammatory cytokine production and impairs repair and corticosteroid responsiveness in lung epithelium
Source: Sci Rep. 2019 Oct 21;9:15047. doi: 10.1038/s41598-019-51517-x (PMC6803636; doi:10.1038/s41598-019-51517-x)
Supplement: Supplementary file 1 — Supplementary methods and figure 1 [file 41598_2019_51517_MOESM1_ESM.docx]

**Online supplementary information**

**Mitochondrial dysfunction increases pro-inflammatory cytokine production and impairs repair and corticosteroid responsiveness in lung epithelium**

*R.F. Hoffmann, M.R. Jonker, S.M. Brandenburg , H.G. de Bruin , N.H.T. ten Hacken, A J.M. van Oosterhout, I.H. Heijink*

**Material and Methods**

***Cell culture***

Wild type A549 and mitochondria-depleted A549 Rho-0 cells were cultured in  Dulbecco's Modified Eagle's medium (DMEM, Sigma, St. Louis, MO, (D6429-500ML) supplemented with MEM Amino Acids (50x) solution (Sigma, M7020-100ML), MEM Non-essential Amino Acid Solution, (Sigma, M7145-100ML), vitamins, (Sigma, M6895-100ML) sodium pyruvate, uridine 50 ng/ml (Sigma, cat. n. U-3003), 2,5 µg/ml amphotericin (Sigma-A2942-100ML), 25% foetal bovine serum (FBS; Hyclone, Logan, UT), 100 U/ml penicillin and 100 µg/ml streptomycin (Invitrogen (Gibco), Breda, The Netherlands) in uncoated T25 flasks and wells. Cells were seeded in a density of 50.000/well in uncoated 24 well plates, grown to confluence and serum-deprived overnight.

***Western blotting***

Cell lysates were prepared and immunodetection was performed as described1 previously^1^ using anti-p-Akt (Cell Signalling Technology, Danvers MA, USA) and anti-actin (Santa Cruz Biotechnology, Santa Cruz, CA) as loading control. Densitometry was performed using the gel-scan program QuantityOne.

Blots were not cropped from different parts of the same gel or from different gels or shown at high-contrast (overexposure).

**A549 wt**

**A549 Rho-0**

**← Total (GAPDH)**

**← p-Akt (~ 65 kD)**


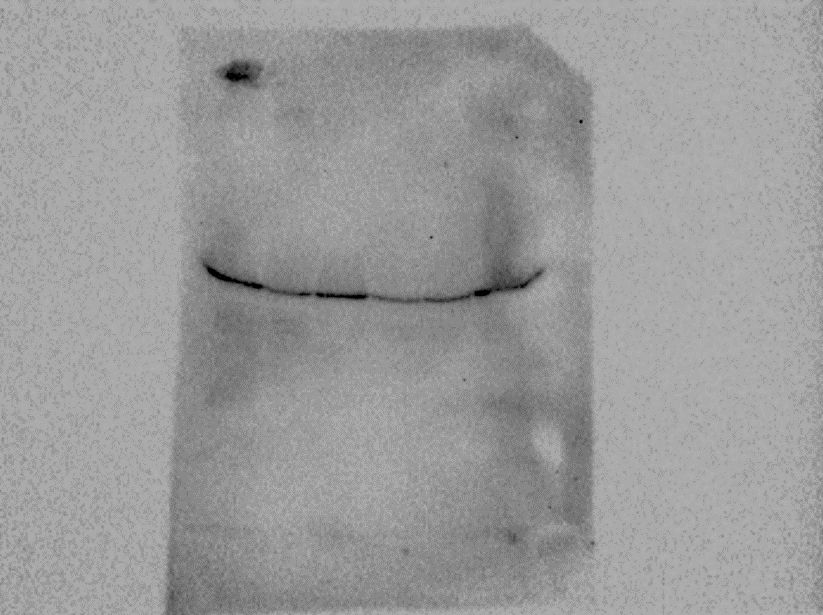

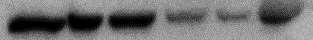


**Figure 1.** **Levels of activated Akt in A549 Rho-0 cells and wild-type A549 cells.** A549 wild-type and Rho-0 cells were grown to confluence and serum deprived for 24 hours. Total cell lysates were prepared and phospho-Akt was detected by western blotting. Actin was used as loading control. Densitometry was performed and levels were related to actin levels. A representative blot and p-Akt/actin ratios (mean±SEM) are depicted (*n=3 per group*). of gels/blots is discouraged*=p=0.05 as tested by the one-tailed Mann-Whitney test.

**References**

1) Heijink, I. H. *et al.* Altered beta2-adrenergic regulation of T cell activity after allergen challenge in asthma. *Clin. Exp. Allergy* **34**, 1356–63 (2004).
